# Identification of host genomic biomarkers from multiple transcriptomics datasets for diagnosis and therapies of SARS-CoV-2 infections

**DOI:** 10.1371/journal.pone.0281981

**Published:** 2023-03-13

**Authors:** Bandhan Sarker, Md. Matiur Rahaman, Md. Ariful Islam, Muhammad Habibulla Alamin, Md. Maidul Husain, Farzana Ferdousi, Md. Asif Ahsan, Md. Nurul Haque Mollah

**Affiliations:** 1 Faculty of Science, Department of Statistics, Bangabandhu Sheikh Mujibur Rahman Science and Technology University, Gopalganj, Bangladesh; 2 Department of Statistics, Bioinformatics Laboratory (Dry), University of Rajshahi, Rajshahi, Bangladesh; 3 Liangzhu Laboratory, Zhejiang University Medical Center, Zhejiang University, Hangzhou, Zhejiang, China; Alagappa University, INDIA

## Abstract

The pandemic of COVID-19 is a severe threat to human life and the global economy. Despite the success of vaccination efforts in reducing the spread of the virus, the situation remains largely uncontrolled due to the random mutation in the RNA sequence of severe acute respiratory syndrome coronavirus 2 (SARS-CoV-2), which demands different variants of effective drugs. Disease-causing gene-mediated proteins are usually used as receptors to explore effective drug molecules. In this study, we analyzed two different RNA-Seq and one microarray gene expression profile datasets by integrating EdgeR, LIMMA, weighted gene co-expression network and robust rank aggregation approaches, which revealed SARS-CoV-2 infection causing eight hub-genes (HubGs) including HubGs; *REL*, *AURKA*, *AURKB*, *FBXL3*, *OAS1*, *STAT4*, *MMP2* and *IL6* as the host genomic biomarkers. Gene Ontology and pathway enrichment analyses of HubGs significantly enriched some crucial biological processes, molecular functions, cellular components and signaling pathways that are associated with the mechanisms of SARS-CoV-2 infections. Regulatory network analysis identified top-ranked 5 TFs (SRF, PBX1, MEIS1, ESR1 and MYC) and 5 miRNAs (hsa-miR-106b-5p, hsa-miR-20b-5p, hsa-miR-93-5p, hsa-miR-106a-5p and hsa-miR-20a-5p) as the key transcriptional and post-transcriptional regulators of HubGs. Then, we conducted a molecular docking analysis to determine potential drug candidates that could interact with HubGs-mediated receptors. This analysis resulted in the identification of top-ranked ten drug agents, including Nilotinib, Tegobuvir, Digoxin, Proscillaridin, Olysio, Simeprevir, Hesperidin, Oleanolic Acid, Naltrindole and Danoprevir. Finally, we investigated the binding stability of the top-ranked three drug molecules Nilotinib, Tegobuvir and Proscillaridin with the three top-ranked proposed receptors (AURKA, AURKB, OAS1) by using 100 ns MD-based MM-PBSA simulations and observed their stable performance. Therefore, the findings of this study might be useful resources for diagnosis and therapies of SARS-CoV-2 infections.

## Introduction

The Severe Acute Respiratory Syndrome Coronavirus 2 (SARS-CoV-2), a highly contagious virus, has resulted in significant loss of human life. It first emerged in Wuhan, Hubei, China in December 2019, and rapidly spread throughout the world. The World Health Organization (WHO) has declared this outbreak a pandemic for the human community [1]. The global healthcare system has been tarnished by this pandemic. As per the WHO report, as of 23 September 2022, there have been 6,512,438 reported fatalities out of a total of 611,421,786 confirmed SARS-CoV-2 infections worldwide. Clinical investigations characterized SARS-CoV-2 infections as acute respiratory tract infections with versatile symptoms, including fever, cough, fatigue, shortness of breath and pneumonia [2]. Despite the fact that the symptoms of SARS-CoV-2 infections are almost known, preventive cures for SARS-CoV-2 infections are not yet at a satisfactory level [3–6]. Early detection of SARS-CoV-2 infections and its treatment with effective drugs may play a vital role to control its outspread [7,8]. Despite the availability of a variety of vaccines against SAR-CoV-2, including those from Pfizer, CoronaVac, BBIBP-CorV, AstraZeneca, BBV152, Moderna, Sputnik, EpiVacCorona, Ad5-nCoV, and WIBP [1,2], scientists and virologists around the world are anxious yet about their effectiveness due to the unstable virus RNA sequence patterns. So, they are continuing their research to understand the molecular mechanism of SARS-CoV-2 infections more clearly for finding effective cures. SARS-CoV-2 infections are developed with the mechanisms of genetic factors and host immune responses [9–11]. Thus, exploring the significant genomic biomarkers, underlying pathogenetic mechanisms and associated drug agents may hold the potential to provide a comprehensive understanding of SARS-CoV-2 infections, and ultimately leading to the discovery of efficacious diagnostic and therapeutic strategies.

Diseases-causing genes are widely used to explore pathogenetic processes and effective drug molecules. Several individual studies explored SARS-CoV-2 infections causing host genomic biomarkers, and their pathogenetic processes based on a single transcriptomics dataset [5,6,12–16]. We reviewed their articles and did not find any common infection-causing genes. Nevertheless, difficulties may arise during the plan to take standard treatment for all against infections of SARS-CoV-2 based on their infection-causing uncommon gene-guided drugs. Therefore, more representative SARS-CoV-2 infections causing genes must be explored for diagnosis and therapies.

Advanced high-throughput technologies are now producing large-scale transcriptome data (RNA-Seq and microarray). So, it has required novel procedures to figure out the consequential information. Integrated bioinformatics and statistical approaches are widely used to develop a novel pipeline for selecting more representative diseases causing genes [17,18]. Weighted gene co-expression network analysis (WGCNA) and robust rank aggregation (RRA) are two powerful cross-validation procedures for exploring the unseen interaction between insight of gene modules and gene samples [19–21]. Therefore, in this study, an attempt was made to explore (i) more representative SARS-CoV-2 infections causing key genes from a transcriptomics profile by cross-validation with the other two independent transcriptomics profiles, (ii) pathogenetic processes and regulatory components of key genes and (iii) key genes guided potential candidate drug agents for the treatment against infections of SARS-CoV-2.

## Materials and methods

**Fig 1:**
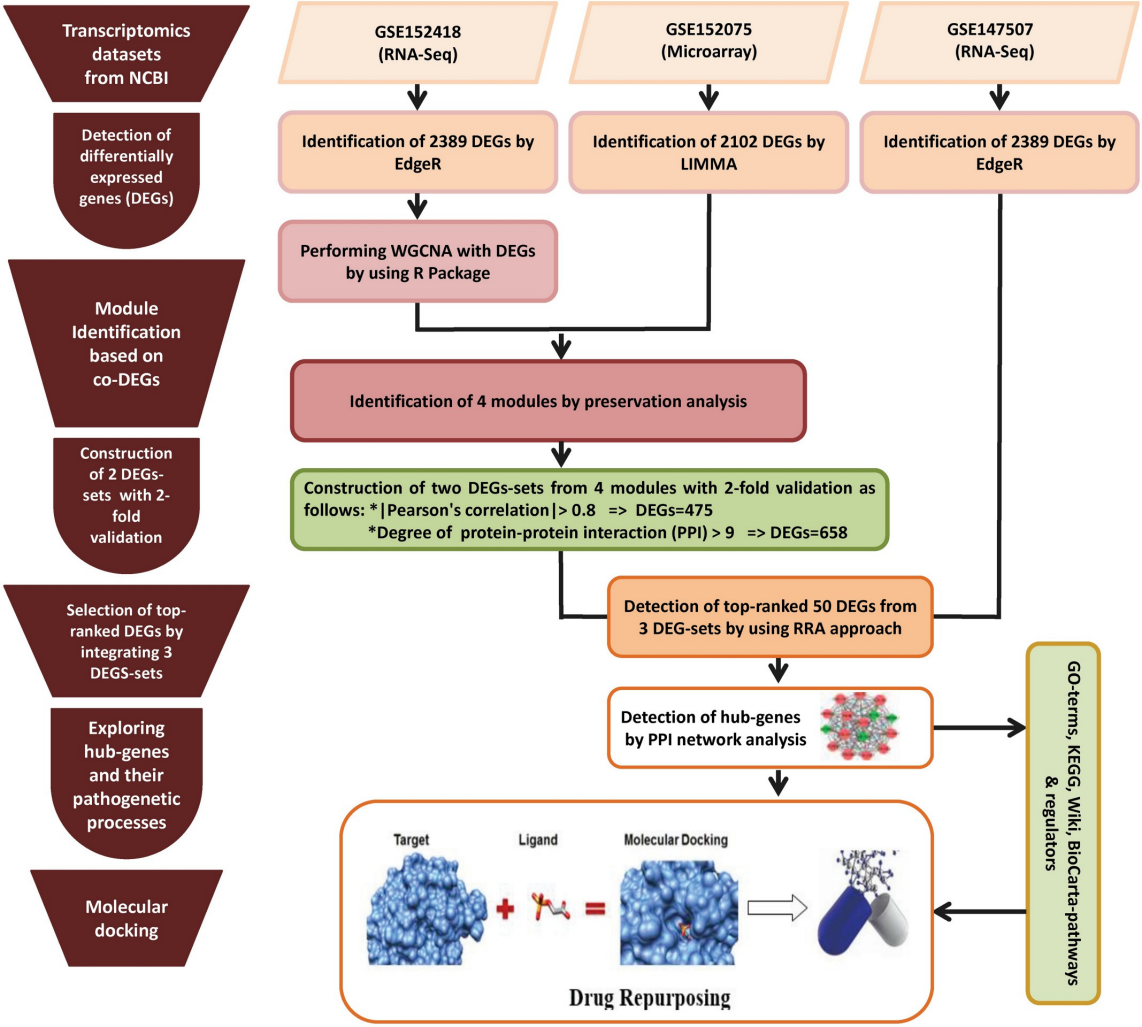
Workflow of the study.

This study analyzed three transcriptomics datasets and associated meta-data on SARS-CoV-2 infections that are freely available in online sources by using integrated statistics and bioinformatics approaches. The workflow of this study is displayed in **Fig 1** and described in the following sections.

### Dataset acquisition and preprocessing

In this study, two RNA-Seq count datasets (GSE152418 and GSE147507), and one microarray dataset (GSE152075) of SARS-CoV-2 (COVID-19) were downloaded from the publicly available gene expression omnibus (GEO) database. GSE152418 raw count data contained 17 COVID-19 and 17 healthy samples [14,22]. GSE147507 raw count data contained 6 samples (3 COVID-19 patients and 3 NHBE samples) [15,23]. GSE152075 is a microarray data containing 430 COVID-19 infected samples and 54 negative samples [16,24–26]. The microarray dataset GSE152075 was downloaded using the Bioconductor package *GEOquery*, and the batch effect of this dataset was removed by using the R package *sva* via Combat_seq [27,28]. GSE152418 is used as a discovery dataset analyzed by WGCNA, GSE147507 is used as an independent validation, and GSE152075 is used as a test dataset. In the COVID-19 datasets, genes that comprise only zero counts have been removed.

### Identification of differentially expressed genes (DEGs)

Differentially expressed genes (DEGs) were identified from the two RNA-Seq count datasets (GSE152418 and GSE147507) through *edgeR R*-package [16], and a microarray dataset (GSE152075) through *limma* R-package [29]. Genes were selected as DEGs that satisfy the criteria of adjusted P-value (Benjamini-Hochberg) < 0.05 and |log2 (FC) | ≥ 1.

### Weighted gene co-expression network analysis (WGCNA) with DEGs

The WGCNA approach was used for exploring modules (clusters) of highly correlated DEGs, summarizing such modules using the cluster eigengene or an intracluster hub genes, relating clusters to each other and to external sample traits (using eigengene networking), and for detecting cluster membership. We implemented this approach using the WGCNA *R* package [30]. In WGCNA, the *pickSoftThreshold* function was used for fitting soft-thresholding powers *β* over the value of maximum *R*^*2*^. Then adjacency matrix and Topological Overlap Matrix (TOM) were created using TOM similarity. The dissimilarity of TOM (dissTOM) was computed using dissimilarity modules. Modules constructions of DEGs were performed using the *hclust* function from the dissTOM based dynamic cut tree (dendrogram). Different parameters were used for preventing large and small modules *i*.*e*., medium sensitivity (*deepSplit* = 2) and minimum module size (*minClusterSize* = 30). Module eigengene (ME) was used for merging similar modules based on *MEDissThres* = 0.25 function.

### Module analysis for validation of DEGs

To find the significant module of co-expressed DEGs (obtained through the WGCNA by integration of test dataset GSE152075), the module preservation function was used. The *module preservation function* is used to identify whether a module is reproducible and robust across the datasets or not [31]. We considered the module to be preserved if the statistic satisfied above Z summary > 10. It is specified negative correlation between preservation statistic-median rank and module preservation, and there is a positive correlation between the module preservation and Z summary statistic. Then the host DEGs were identified based on 2-fold cross-validation namely module membership statistic (MMS) calculated by the Pearson’s correlation and Protein-protein interaction networks (PPIN). Genes of MMS, PPIN and DEGs from independent datasets were chosen as host signatures by RRA. The final subsets of host hub DEGs were separated by PPIN analysis (Fig 1).

### Protein-protein interaction (PPI) network analysis based on validated DEGs

We performed PPI network analysis to explore SARS-CoV-2 infection causing hub-genes. To construct the PPI network for host signatures, genes data were collected from the STRING database [32], and the Cytoscape software [33] was used to construct the network based on the parameter: confidence score ≥ 0.4 and most extreme interactors = 0 for cutoff models. Similarly, the hub-genes signatures were separated. After that, these hub signatures were used for gene enrichment analysis, finding transcriptional and post transcriptional regulators and drug repurposing with molecular docking analysis described in the next section.

### Functional enrichment analysis of hub-genes

The Gene Ontology (GO), Kyoto Encyclopedia of Genes and Genomes (KEGG), WikiPathways and BioCarta pathway enrichment analyses for hub-genes were performed via the web-based tool *Enrichr* [34] to explore the pathogenetic processes of SARS-CoV-2 infections. P-value (Adjusted) < 0.05 was used to extract the significant biological information.

### Hub-genes regulatory network analysis

To explore transcriptional and post-transcriptional regulators of hub-genes, we performed transcription factors (TFs) versus hub genes and micro RNAs versus hub genes interaction by using the databases TF2DNA [35] and miRDB [36], respectively.

### Meta-data collection

We collected 177 drug agents as a meta-data from the literature review of 16 COVID-19 related articles to explore the potential candidate drugs (S1 Table). To validate the proposed repurposed candidate drugs by using molecular docking (MD) analysis with the top-ranked receptor proteins associated with COVID-19, the metadata were obtained from the literature review (S2 Table). We selected top-ranked COVID-19 associated 8 receptor proteins as meta-data by reviewing 24 newly published articles to assess the binding affinity of the proposed candidate drugs with these receptor proteins (S2 Table).

### Molecular docking

To explore repurposable effective drug molecules for COVID-19 by *in-silico validation*, molecular docking analysis was performed between the target proteins and meta-drug agents. Our proposed HubGs mediated proteins and their associated TFs proteins were considered the drug target receptors, and 177 meta-drugs as the drug-agents that were obtained from the literature review and other sources as mentioned earlier in the data sources (S1 Table). From Protein Data Bank (PDB) [37] and SWISS-MODEL [38], the 3-Dimensional (3D) structures of receptor proteins were downloaded. The PubChem database [39] was used to download the 3D structures of drug agents. The PyMOL 2.4.1 software was used to visualize the 3D structure of the target receptor proteins [40]. The protein chains which were not a part of the gene are deleted [41]. Then, Swiss PDB viewer software was used to add charges and minimize the energy of the target proteins [42]. The target proteins were prepared for molecular docking analysis by eliminating water molecules and ligand heteroatoms, adding polar hydrogens, and converting them to pdbqt format using AutoDock tools 1.5.7 [43]. Avogadro software was used for minimizing the energy of the ligands [44]. The ligands were prepared for dynamic simulation by setting the torsion tree and rotatable, and nonrotatable bonds present in the ligand through AutoDock tools 1.5.7 [43]. Then, the binding affinities score between the ligand and receptors were calculated by using AutoDock Vina [45]. The Discovery Studio Visualizer 2019 was used to analyze the docked complexes. Let ***S***_*ij*_ indicates the binding score of *i*^th^ receptors (*i* = 1, 2, …, *m*) with the *j*^th^ ligand (*j* = 1, 2,…, *n*). Then receptors were ordered according to the decreasing order of row means $\sum_{j=1}^{n}S_{ij}/n;\;i=1,2,\ldots ,m$ and ligands were ordered according to the decreasing order of column means $\sum_{i=1}^{m}S_{ij}/m;\;j=1,2,\ldots ,n$ to select the top-order ligands as the candidate drug agents [5,17,46].

### Molecular Dynamics (MD) simulation

To perform the dynamic properties of top-ordered protein-ligand complexes, YASARA [47] and the AMBER14 force field [48] were used in Molecular Dynamics (MD) simulations. We assigned the ligands parameters for the complexes by using AutoSMILES [49] algorithms, which automatically parameterize unknown organic molecules by computing semi-empirical AM1 Mulliken point charges with the COSMO solvation model, assigning AM1BCC [50] atom and bond types, and assigning general AMBER force field (GAFF) [51] atom types, and the remaining parameters of force field. In a simulation cell, the hydrogen bonding network of protein-ligand complexes were optimized and solvated by a TIP3P [52] water model before the simulation. We considered the solvent density of 0.997 g L^-1^ to maintain the periodic boundary conditions. During solvation, titratable amino acids in the protein complex were assigned to calculate pKa. The initial energy minimization process of each simulation system, consisting of 53735, 54335, and 79559 atoms for AURKA *vs*. Nilotinib, AURKB *vs*. Tegobuvir, and OAS1 *vs*. Proscillaridin complexes was performed by a simulated annealing method, respectively using the steepest gradient approach (5000 cycles).

A multiple-time-step algorithm [53] with 2.50 fs time step interval under physiological conditions (298 K, pH 7.4, 0.9% NaCl) [54] was used to run the simulation of each complex. The linear constraint solver (LINCS) [55] algorithm was used to constrain all bond lengths, and SETTLE [56] was employed to control the water molecules. PME methods [57] were used to describe long-range electrostatic interactions, and 100 ns MD simulation was performed at Berendsen thermostat [58] and constant pressure. The trajectories were captured at every 250 ps for further analysis, and subsequent analysis was performed by the built-in script of the YASARA [59] macro and SciDAVis software (http://scidavis.sourceforge.net/). All the captured snapshots were used to calculate MM-Poisson–Boltzmann Surface Area (MM-PBSA) binding free energy by YASARA software using the formula below [60]:

$$\mathrm{Binding}\;\mathrm{free}\;\mathrm{energy}=(E_{potReceptor}+E_{solvReceptor}+E_{potLigand}+E_{solvLigand})—(E_{potComplex}+E_{solvComplex})$$

Here, we computed MM-PBSA binding energy by YASARA default macros using AMBER 14 as a force field, with larger positive energies indicating better binding [61].

## Results

### Identification of DEGs

Two different raw RNA-Seq datasets (GSE152418 and GSE147507) and one microarray gene expression profile (GSE152075) were used for differential expression analysis (Table 1). We identified 2389 DEGs with 636 up-regulated and 1753 down-regulated genes for the GSE152418 dataset, and 540 DEGs with 213 up-regulated and 327 down-regulated genes for the GSE147507 dataset. We also identified 2102 DEGs with 570 up-regulated and 1532 down-regulated genes for GSE152075. These identified DEGs are used for further analysis.

**Table 1:** Data description.

Datasets	DE genes		Total samples	COVID-19 samples	Normal samples
	Up- regulated	Down- regulated			
GSE152418	636	1753	34	17	17
GSE147507	213	327	6	3	3
GSE152075	570	1532	484	430	54

### WGCNA analysis for validation of DEGs

**Fig 2:**
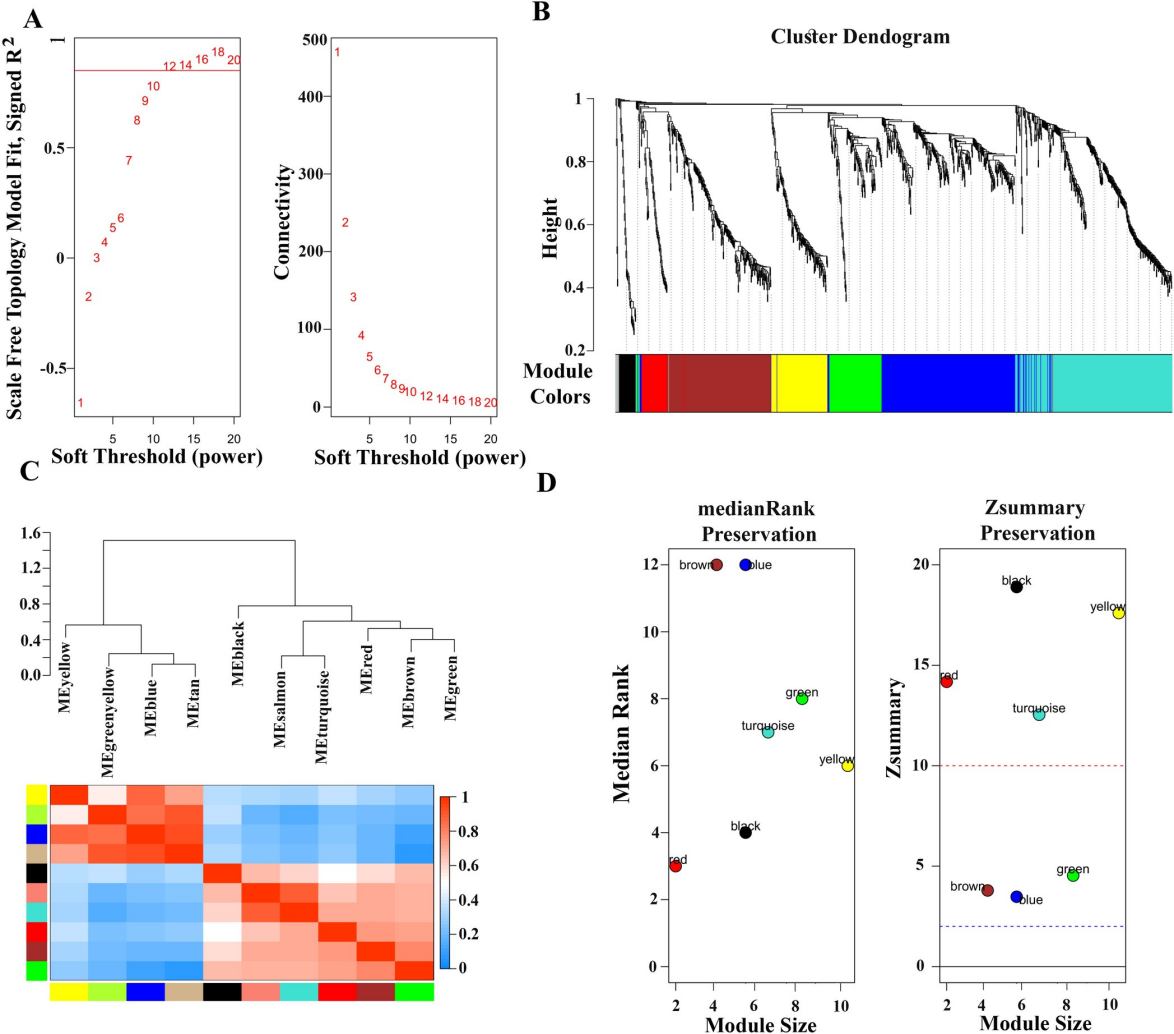
Cross-validation of DEGs by WGCNA. (A) Illustration of soft-thresholding powers based on the scale-free tropology model fit (left) and the mean connectivity (right). (B) The dendrogram of all DEGs clustered based on a dissimilarity measure (1-TOM). (C) The dendrogram of eigengene module and cluster analysis of eigengene network by heatmap summarize the modules yielded in the clustering analysis. (D) Median rank preservation (left) and Z summary preservation (Right); the black, red, yellow and turquoise indicate the strong preservation above dashed lines Z = 2 and Z = 10.

WGCNA was performed with the DEGs in the dataset GSE152418. The power value is the most important parameter which is contaminated with the average connectivity degree (ACD) and independence of co-expression modules (ICEM). The power value (Fig 2A) shows that ACD was greater, and the value of ICEM reached the expected value 0.8. Thus, the power value is ready to create the co-expression module and constructed with multiple colors presented in Fig 2B. The co-expression network constructed seven modules, namely black, red, yellow, turquoise, green, brown and blue, based on the soft threshold power *β* = 6 with *R*^2^ = 0.80. The Eigengene dendrogram and eigengene network heatmap represent the interactions among the co-expression modules (Fig 2C). Then the dataset GSE152418 was compared with the test dataset GSE152075, and the summary of preservation statistic was visualized (Fig 2D). We observed that among the seven modules black, red, yellow and turquoise are the most stable modules (Zsummary statistic: above *Z* = 2 and *Z* = 10). The remaining modules were considered nonstable (Z summary statistic < 10). Black, red, yellow, and turquoise colors showed minimum median rank statistic which indicated that their preservation is best than the other modules.

### Identification of hub-genes from validated DEGs

**Fig 3:**
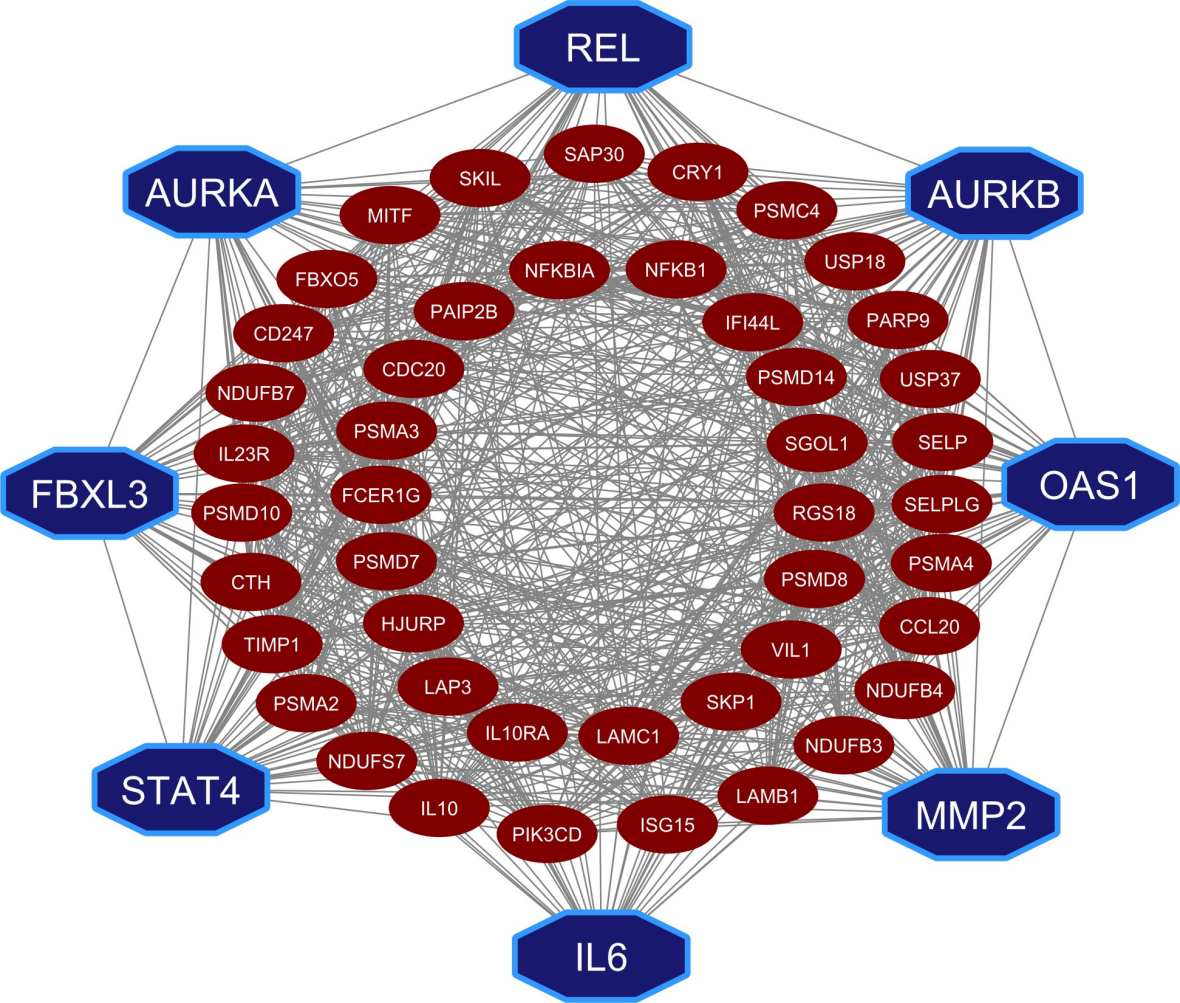
PPI network of validated DEGs to identify hub-genes.

We identified 475 significant genes using high connectivity modules black, red, yellow and turquoise through the threshold |cor.geneModuleMembership| > 0.8. Again, the PPI network extracted 658 significant genes based on the highest degree > 9 for the four modules. These two gene sets were validated using the validation DEGs set. Validation DEGs set obtained from the GSE147507 dataset to confirm the most stable gene set of COVID-19. We used RRA to identify the top 50 significant genes in this case. Finally, eight hub genes (*REL*, *AURKA*, *AURKB*, *FBXL3*, *OAS1*, *STAT4*, *MMP2* and *IL6*) are identified from the top 50 genes through the PPI network analysis (Fig 3).

### Functional enrichment analysis of hub-genes

Various pathway enrichment analyses were performed to explore further biological insight of the HubGs. GO and pathway terms with P-value (adjusted) < 0.05 were considered significant. The information of GO with their three subsections (BPs, MFs, CCs) is presented in Table 2. The significant BPs are mainly enriched in the negative regulation of chemokine production, liver development and response to peptide, etc. The significant MFs enriched in the histone serine kinase activity, histone kinase activity, interleukin-6 receptor binding, etc. The significant CCs are enriched in the spindle microtubule, condensed chromosome, microtubule, etc. Different pathways; KEGG, WikiPathways, and BioCarta analysis results are presented in Table 3. The KEGG pathways for the hub genes are enriched in several pathways such as inflammatory bowel disease, AGE-RAGE signaling pathway in diabetic complications, pathways in cancer and coronavirus disease, etc. The WikiPathways pathway analysis results enhanced in Photodynamic therapy-induced NF-kB survival signaling WP3617, FOXP3 in COVID-19 WP5063, COVID-19 adverse outcome pathway WP4891, STING pathway in Kawasaki-like disease and COVID-19 WP4961, and Host-pathogen interaction of human coronaviruses—interferon induction WP4880, etc. BioCarta is mostly involved in Interleukin-27-mediated signaling events, FRA pathway, Interleukin-23-mediated signaling events and so on.

**Table 2:** Significantly enriched top-ranked 6 GO-terms with hub-genes.

Category	GO-ID	GO-terms	P-values (Adjusted)	Hub-genes
Biological process (BP)	GO:0032682	negative regulation of chemokine production	<0.001	IL6, OAS1
	GO:0001889	liver development	<0.001	IL6, AURKA
	GO:1901652	response to peptide	<0.001	MMP2, STAT4
	GO:0060700	regulation of ribonuclease activity	0.003	OAS1
	GO:0061888	regulation of astrocyte activation	0.003	IL6
	GO:0032466	negative regulation of cytokinesis	0.003	AURKB
Molecular Function (MF)	GO:0035174	histone serine kinase activity	<0.001	AURKA, AURKB
	GO:0035173	histone kinase activity	<0.001	AURKA, AURKB
	GO:0005138	interleukin-6 receptor binding	0.009	IL6
	GO:0004674	protein serine/threonine kinase activity	0.018	AURKA, AURKB
	GO:0070566	adenylyltransferase activity	0.018	OAS1
	GO:0004222	metalloendopeptidase activity	0.041	MMP2
Cellular Component (CC)	GO:0005876	spindle microtubule	0.002	AURKA, AURKB
	GO:0005874	microtubule	0.007	AURKA, AURKB
	GO:0005819	spindle	0.007	AURKA, AURKB
	GO:0000779	condensed chromosome, centromeric region	0.009	AURKB
	GO:0015630	microtubule cytoskeleton	0.013	AURKA, AURKB
	GO:0043231	Intracellular membrane-bounded organelle	0.032	OAS1, REL, FBXL3, AURKA, AURKB

**Table 3:** Significantly enriched top-ranked 6 biological pathways with hub-genes in different databases.

Databases	Pathways	P-values (Adjusted)		Hub-genes
KEGG	Inflammatory bowel disease		0.003	IL6, STAT4
	AGE-RAGE signaling pathway in diabetic complications		0.003	IL6, MMP2
	Pathways in cancer		0.003	IL6, MMP2, STAT4
	Measles		0.003	IL6, OAS1
	Influenza A		0.003	IL6, OAS1
	Coronavirus disease		0.004	IL6, OAS1
WikiPathways	Photodynamic therapy-induced NF-kB survival signaling WP3617		<0.001	IL6, MMP2, REL
	miRNAs involvement in the immune response in sepsis WP4329		<0.001	IL6, REL
	Interferon type I signaling pathways WP585		<0.001	REL, STAT4
	FOXP3 in COVID-19 WP5063		0.007	IL6
	COVID-19 adverse outcome pathway WP4891		0.007	IL6
	STING pathway in Kawasaki-like disease and COVID-19 WP4961		0.009	REL
	Host-pathogen interaction of human coronaviruses—interferon induction WP4880		0.013	OAS1
BioCarta	Interleukin-27-mediated signaling events		<0.001	IL6, STAT4
	FRA pathway		<0.001	IL6, MMP2
	Interleukin-23-mediated signaling events		<0.001	IL6, STAT4
	Aurora B signaling		<0.001	AURKB, AURKA
	Alpha-M beta-2 integrin signaling		<0.001	IL6, MMP2
	FOXM1 transcription factor network		<0.001	MMP2, AURKB

### Hub-genes regulatory network analysis

**Fig 4:**
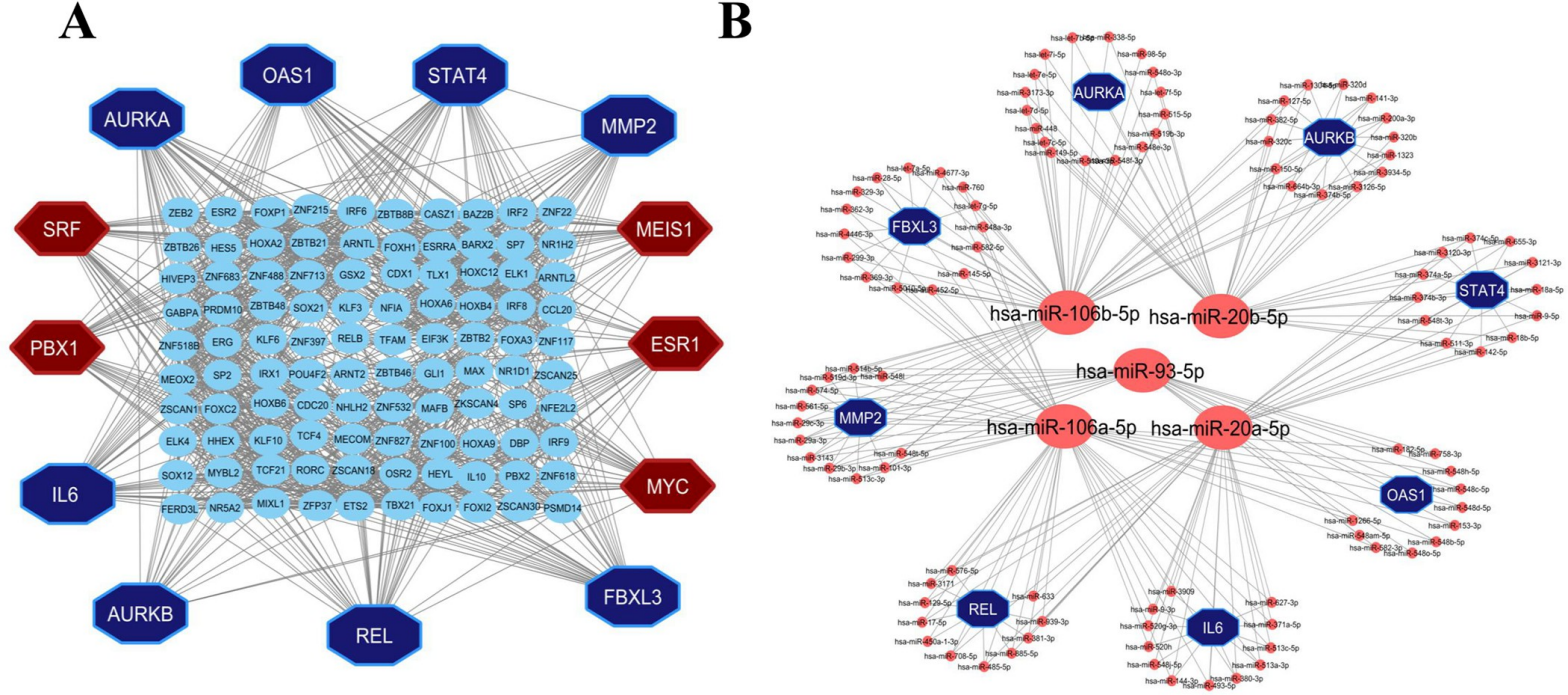
Hub-genes regulatory network analysis with (A) Transcription factors (TFs) and (B) micro RNAs (miRNAs).

We identified SRF, PBX1, MEIS1, ESR1 and MYC hub-TFs (Fig 4A), and hsa-miR-106b-5p, hsa-miR-20b-5p, hsa-miR-93-5p, hsa-miR-106a-5p and hsa-miR-20a-5p hub-miRNAs (Fig 4B) from the TFs-HubGs and miRNA-HubGs interaction network, respectively.

### Exploring candidate drugs by molecular docking simulation

We considered HubGs mediated 8 proteins (REL, AURKA, AURKB, FBXL3, OAS1, STAT4, MMP2 and IL6) and their regulatory 5 hub-TFs proteins (SRF, PBX1, MEIS1, ESR1 and MYC) as the receptor proteins. The 3D structure of REL, AURKA, AURKB, FBXL3, OAS1, STAT4, MMP2, IL6, SRF, PBX1, MEIS1, ESR1 and MYC; targets were downloaded from PDB (Protein Data Bank) using the codes 1a3q, 6gra, 3af3, 4i6j, 4ig8, 4gj2, 3ayu, 5fuc, 1srs, 1puf, 5ego, 1uom, and 6e16, respectively. Then we considered 177 drug molecules (drug agents) that were selected by the literature review of COVID-19 related articles (S1 Table) and downloaded their 3D structures from the PubChem database. Then we performed molecular docking analysis of each receptor with each agent.

**Fig 5:**
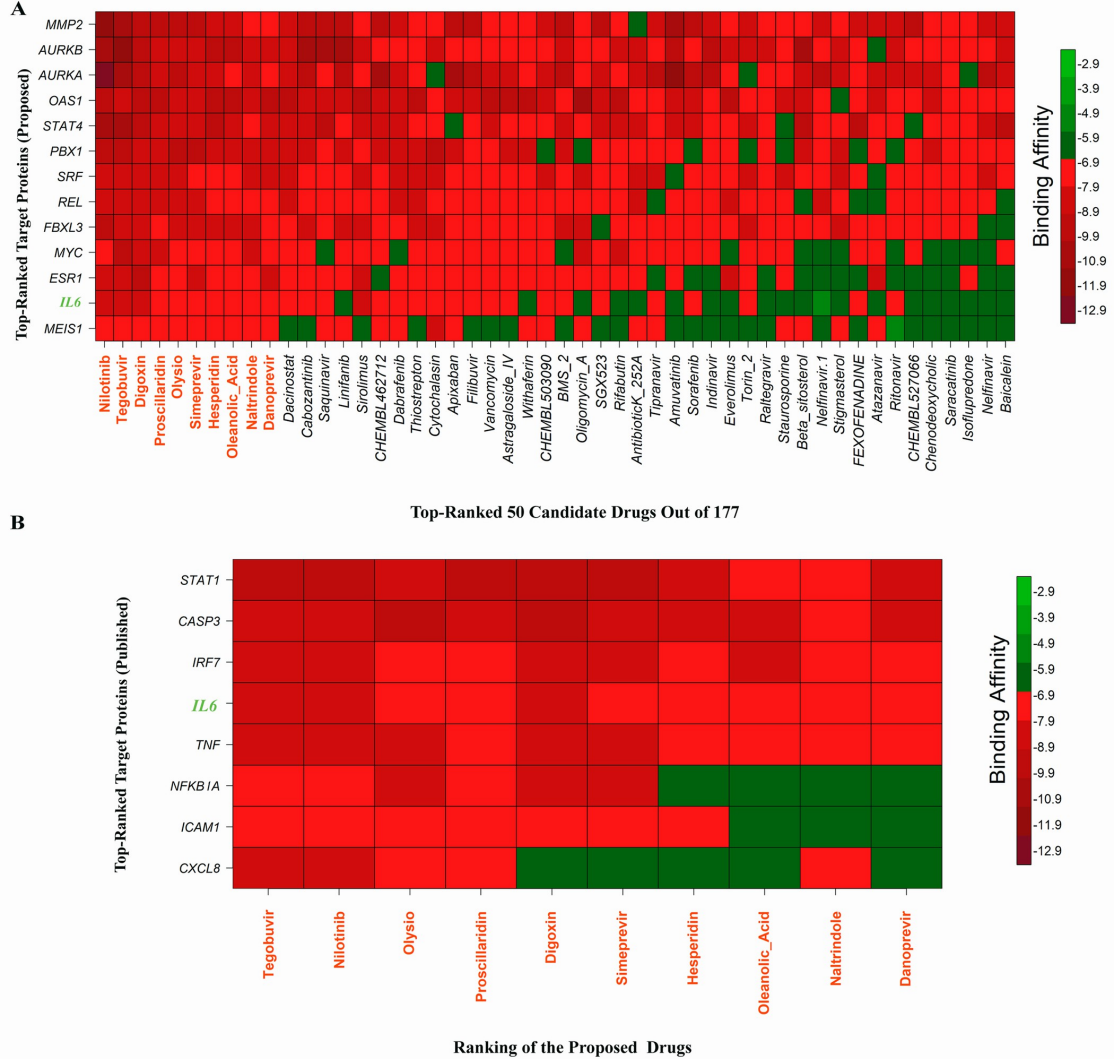
Matrix of binding affinity scores between receptors and ligands computed by molecular docking. (A) Row indicates ordered 13 proposed receptor proteins and column indicates the top-ordered 50 drug agents out of 158, where red colors indicate the strong binding affinities, (B) Row indicates top-ranked 8 receptor proteins obtained through published literature, and column indicates the proposed top-ordered 10 drug agents out of 158, where red colors indicate the strong binding affinities.

**Fig 6:**
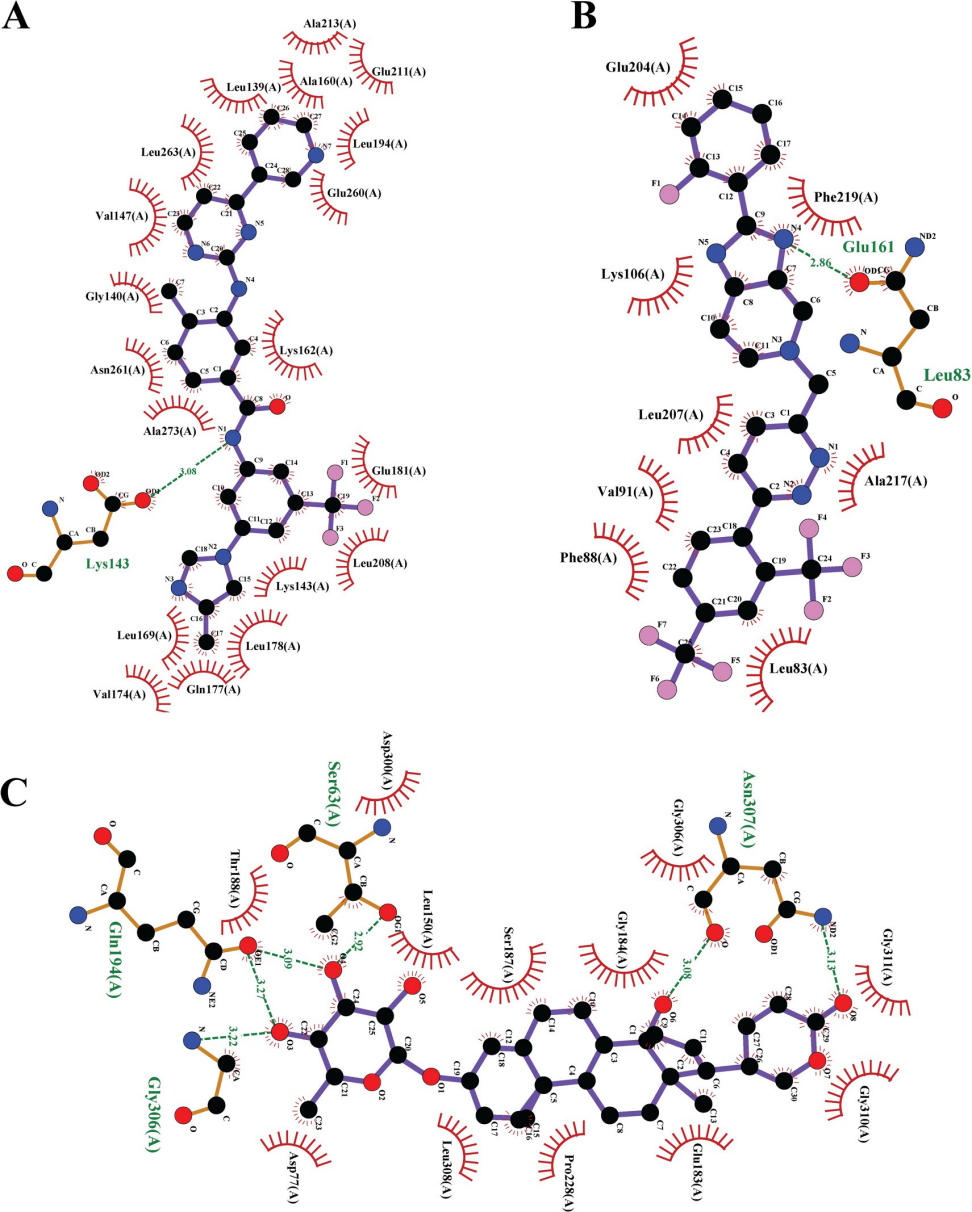
The 2D view of strong binding interactions between targets and drugs are shown by Ligplot. (A) AURKA *vs*. Nilotinib, (B) AURKB *vs*. Tegobuvir, and (C) OAS1 *vs*. Proscillaridin.

**Fig 7:**
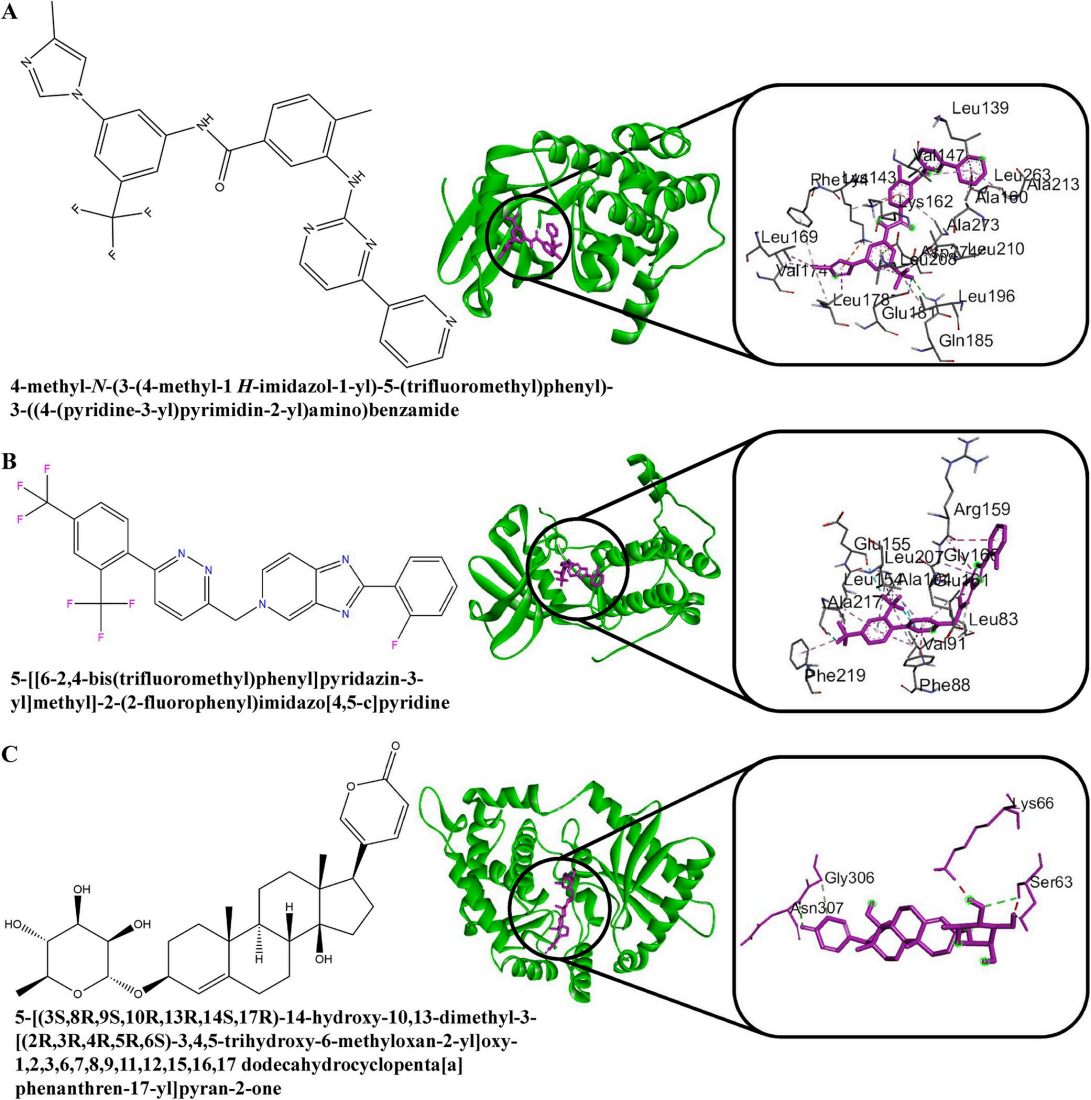
Lead compound (left side) and three complexes of three-dimensional chemical interactions (right side) obtained from molecular docking. (a) AURKA *vs*. Nilotinib, (b) AURKB *vs*. Tegobuvir, and (c) OAS1 *vs*. Proscillaridin.

Fig 5A displayed the binding affinity score matrix between the ordered receptors and drug agents. We observed that each of the top 10 lead compounds (Nilotinib, Tegobuvir, Digoxin Proscillaridin, Olysio, Simeprevir, Hesperidin, Oleanolic Acid, Naltrindole and Danoprevir) produces binding affinity scores less than or equal to -7.0 kcal/mol with all of our suggested receptors (Section-I in S1 File for score matrix). Therefore, we considered these 10 drugs as the candidate drug agents in this study. To validate the proposed drugs against the state-of-the-art alternative independent receptors, we considered the top-ranked 8 hub-genes **(***CASP3*, *CXCL8*, *ICAM1*, *IL6*, *NFKBIA*, *STAT1*, *TNF* and *IRF7*) that are common in at least 3 articles (S2 Table) out of 24. The 3D structures of these 8 independent receptor proteins were downloaded from Protein Data Bank (PDB) with codes 4ps0, 6n2u, 5mza, 5fuc, 1nfi, 1bf5, and 7kba, and receptor protein IRF7 were retrieved from the SWISS-MODEL using the UniProt IDs Q92985, respectively. Fig 5B represents the binding affinities (kcal/mol) between the proposed drugs and publicly available top-ranked independent receptors. We observed that three lead compounds (lead1: Tegobuvir, lead2: Nilotinib, lead3: Proscillaridin) strongly bind with all independent receptors (Section-II in S1 File for score matrix). Table 4 represents the summary results of interacting properties of the top targets with top-ranked lead compounds that produced highest binding scores. We also examined their complete interaction profile including hydrophobic, hydrogen bonds, and electrostatic interactions. We illustrated 2D structure of proteins and ligands interaction in Fig 6. The 3D structure of their interacting complex and top-ranked lead compounds are shown in the Fig 7. To investigate the stability of the top three complexes, we performed molecular dynamic simulations as discussed in the next section.

**Table 4:** Docking results of interacting proteins and drugs. The last row shows key interactions of amino acids and their binding types with potential targets.

**Potential Targets**		AURKA	AURKB	OAS1
**Potential Ligands**		Nilotinib	Tegobuvir	Proscillaridin
**Binding Affinity (kcal/ mol)**		-12	-11	-9.8
**Interacting Amino Acids**	Hydrogen Bond	LYS143	LEU83, GLU161	SER63, ASN307, GLN 194, GLY306
	Hydrophobic Interactions	LEU169, LEU263, LEU164, ALA273, ALA213, LYS143, VAL147, ALA160, LEU194, GLU211, LEU139, GLU260, GLY140, LYS162, ASN261, GLU181, LEU208, LEU178, GLN177, VAL174	GLU204, LYS106, PYS106, PHE219, LEU207, ALA217, VAL91, LEU154, PHE88, LEU83,	PRO228, LEU308, TYR230, ASP300, THR188, ASP77, LEU308, LEU150, SER187, GLY184, GLY306, GLY311, GLY310, GLN 183
	Electrostatic	LYS143	-	-

### Molecular Dynamic (MD) simulations

**Fig 8:**
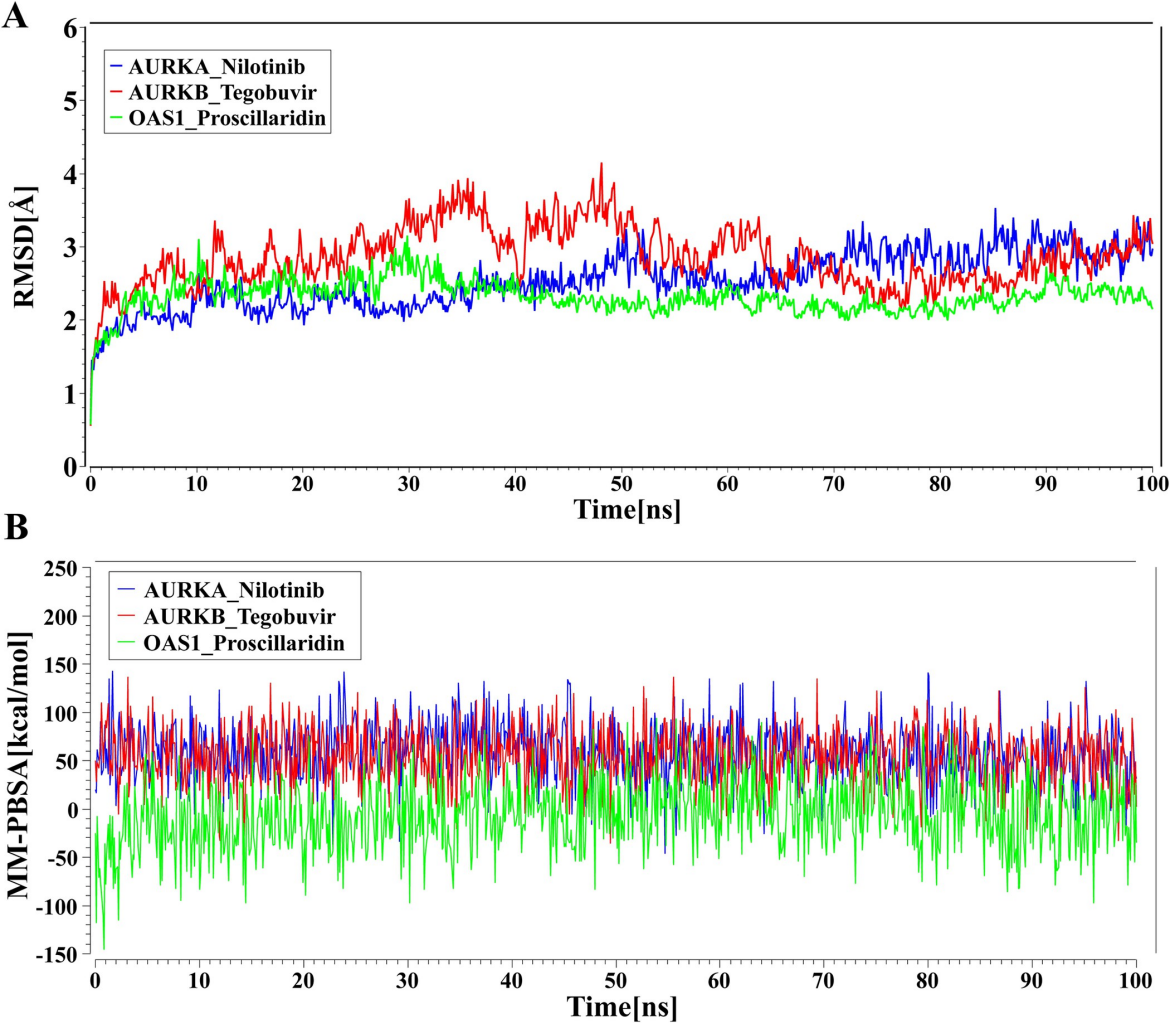
MD simulations of top-ranked three complexes. (A) Time evolution of RMSDs for each of the top-ranked three complexes. (B) Binding free energy (kcal/mol) of each snapshot was calculated by MM-PBSA, representing the change in binding stability of each complex during simulations; positive values indicate better binding. Complexes: blue AURKA *vs*. Nilotinib, red AURKB *vs*. Tegobuvir, and green. OAS1 *vs*. Proscillaridin.

Three predicted drug agents (Nilotinib, Tegobuvir, and Proscillaridin) showed the highest binding affinities with AURKA, AURKB, and OAS1 proteins, respectively (Table 4). Therefore, three complexes (AURKA *vs*. Nilotinib, AURKB *vs*. Tegobuvir, and OAS1 *vs*. Proscillaridin) were considered for stability analysis using 100 ns MD-based MM-PBSA simulations. We observed that these 3 complexes (AURKA *vs*. Nilotinib, AURKB vs Tegobuvir, OAS1 *vs*. Proscillaridin) showed significant stability between the variations of moving and initial drug-target protein complexes (Fig 8A). RMSD values corresponding to each complex were calculated. All the systems projected the RMSD values around 1.5 Å to 4.15 Å. The average RMSD values for AURKA *vs*. Nilotinib, AURKB *vs*. Tegobuvir, and OAS1 *vs*. Proscillaridin complexes were 2.543 Å, 2.863 Å, and 2.324 Å, respectively. The OAS1 *vs*. Proscillaridin complex displayed a more rigid conformation than the other complexes, reached equilibrium at 35 ns, and remained almost stable, after that AURKA *vs*. Nilotinib complex showed almost stable performance during 10 ns to 35 ns, 52 ns to 70 ns and the remaining times there were irregular fluctuations in the RMSD. On the contrary, AURKB *vs*. Tegobuvir complexes exhibited irregular fluctuation and RMSD values fluctuate from 2.0 Å to 4.15 Å over the time period. In addition, MM-PBSA binding energy for three complexes were also calculated, and Fig 8B illustrated the binding energies of the complexes. On average, AURKA *vs*. Nilotinib, AURKB *vs*. Tegobuvir, and OAS1 *vs*. Proscillaridin complexes produced MM-PBSA binding energies 58.5 kcal/mol, 53.92 kcal /mol, and– 6.834 kcal /mol, respectively.

## Discussion

**Fig 9:**
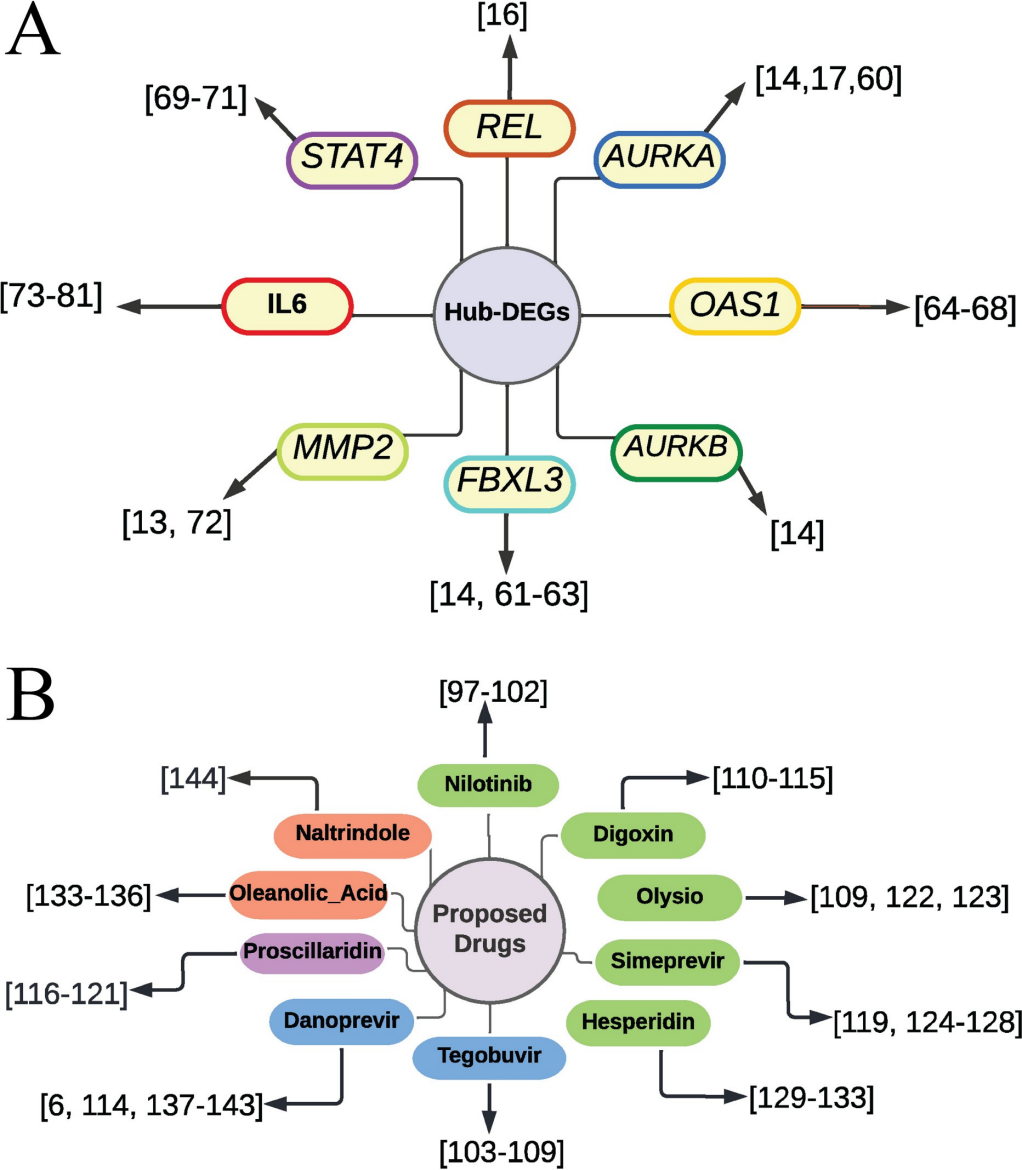
Validation of hub genes and candidate drugs in favor of SARS-CoV-2 by the literature review. (A) Validation of the proposed HubGs: circles with node color indicates hub genes, and each connected network with number(s) indicates the reference(s) of gene(s) of SARS-CoV-2 (B) Validation of the proposed candidate drugs: circles with green color indicate FDA approved, light blue color indicates investigational drugs and purple color indicates experimental drugs and red color indicates unapproved drugs, and each connected network with number(s) indicates the reference(s) of drug(s) of SARS-CoV-2.

COVID-19 is the most recent and ongoing pandemic that has adversely affected on human health and the world’s economy. Though vaccination programs were started globally at a marginal rate, it is still a threat to public health. Gene signatures are the pathological indicator for describing diseases at a molecular level. In this study, we used bioinformatics approach to detect gene signatures and potential therapeutic drugs for the treatment of COVID-19 patients. The present study employed three different datasets (Table 1) to identify potential DEGs between COVID-19 and control samples. The results of the analysis revealed a total of 2389 and 540 DEGs from two RNA-Seq datasets and 2102 DEGs from the microarray dataset. To select the potential DEGs, we validated these 3 DEGs-sets by WGCNA, PA and RRA procedures. Then we selected top-ranked 50 DEGs as the most potential DEGs. We performed protein-protein interaction (PPI) network analysis of those 50 DEGs to select the HubGs. Finally, we selected top-ranked 8 DEGs (*REL*, *AURKA*, *AURKB*, *FBXL3*, *OAS1*, *STAT4*, *MMP2*, *IL6*) as the HubGs (Fig 3), that were used for further investigation of SARS-CoV-2 infections. The literature review also supported these HubGs as the SARS-CoV-2 infection-causing genes (Fig 9A). As for example, the gene *REL* has been previously reported as a hub gene for SARS-CoV-2 infections [16]. By combining some studies, we found that the gene *AURKA* is a common targeted protein for both COVID-19 and lung adenocarcinoma patients [14,17,62]. The gene *AURKB* plays a crucial role as a biomarker gene in the diagnosis and prognosis of COVID-19 patients [14]. The gene *FBXL3* has been identified as a core gene of COVID-19 [14,63–65]. It has been noted that the gene *OAS1* is an important gene influencing COVID-19 patients [66–70]. The gene *STAT4* is the human transcriptomic factor of COVID-19 [71–73]. The gene *MMP2* has been recognized as a hub gene in COVID-19-infected patients [13,74]. The gene *IL-6* can safeguard against basic circumstances with coronavirus, diminishing IL-6 articulation [75–83]. The interaction network analysis between HubGs and transcription factors (TFs) revealed the top-ranked 5 TFs genes (SRF, PBX1, MEIS1, ESR1 and MYC*)* as the key transcriptional regulators of HubGs (Fig 4A). Notably, the SRF gene demonstrated a unique and dysfunctional pattern in COVID-19 [5,6,84,85]. The TF genes PBX1 has been found to possess multiple functions relevant to cell development and has been associated with tumor agents and COVID-19 [16,86], MEIS1 has been identified as the targeted agent of SARS-CoV-2 [16,87], ESR1 has been noted to act as an antiviral signature that disrupts the viral membrane of the SARS-CoV-2 protein [88]. Furthermore, MYC is another target gene of COVID-19, has been reported to have various functions, including regulation of chromatin sites, modulation of cellular metabolism, and versatility across various cell types [87,88]. The hub-genes versus micro-RNA interaction network analysis revealed top-ranked 5 miRNAs (hsa-miR-106b-5p, hsa-miR-20b-5p, hsa-miR-93-5p, hsa-miR-106a-5p and hsa-miR-20a-5p) as the post-transcriptional regulators of hub-genes (Fig 4B). The miRNA, hsa-miR-106b-5p, has been identified as a tumor promoter and targeted receptor for different cancers [89]. The hsa-miR-20b-5p miRNA has been shown to play an antiviral role in patients infected with SARS-CoV and SARS-CoV-2, as well as the up-regulated signature of the influenza virus [90]. The hsa-miR-93-5p miRNA is associated with human cancerous growth and encourages angiogenic operation [91]. The miRNA, hsa-miR-106a-5p, promotes virus mechanism of COVID-19 [92]. The miRNA, hsa-miR-20a-5p has been shown to play a significant role in respiratory viruses including adenovirus 2, influenza A and RSV [93].

To explore the biological insights underlying HubGs we used web-based tool *Enrichr*. Pathological information of HubGs described the significance of biomarker agents by using gene ontology and pathway analysis. GO analysis enriched with the regulation of acute inflammatory response [11], interleukin-6 receptor binding [94], and Intracellular membrane-bounded organelle [95] (Table 2). KEGG pathway associated with influenza A, coronavirus disease [96], bladder cancer and malaria. WikiPathways Interferon type I signaling pathways WP585, FOXP3 in COVID-19 WP5063, COVID-19 adverse outcome pathway WP4891, STING pathway in Kawasaki-like disease and COVID-19 WP4961 (Table 3). To find the effective drug molecules against COVID-19, we used the proposed 8 target proteins and their regulatory 5 key TFs proteins as the receptor proteins. We performed their docking analysis with 177 meta-drug agents (S1 File).

Then we picked up the top-ranked 10 drugs (Nilotinib, Tegobuvir, Digoxin, Proscillaridin, Olysio, Simeprevir, Hesperidin, Oleanolic Acid, Naltrindole, and Danoprevir) as the candidate drug agents based on their strong binding affinities with all the target proteins (Fig 5A). These drug molecules are also supported by other individual studies for the treatment against SARS-CoV-2 infections which includes Nilotinib [97–102], Tegobuvir [103–109], Digoxin [110–115], Proscillaridin [116–121], Olysio [109,122,123], Simeprevir [119,124–128], Hesperidin [129–133], Oleanolic Acid [133–136], Danoprevir [6,114,137–143], Naltrindole [144] for the treatment against COVID-19 (Fig 9B). Fig 5B displays the results of cross-validation of our suggested ten candidate drug agents with the top-ranked independent receptor proteins, and observed their strong binding affinities. Finally, the binding stability of the top three complexes (AURKA vs. Nilotinib, AURKB vs. Tegobuvir, and OAS1 vs. Proscillaridin) were investigated by molecular dynamics (MD) based MM-PBSA simulations, which revealed their stable performance (Fig 8) [145,146].

The phylogenetic tree and pairwise alignment results on identities, similarities and gaps of HubGs (*REL*, *AURKA*, *AURKB*, *FBXL3*, *OAS1*, *STAT4*, *MMP2* and *IL6*) protein sequences showed that AURKA and AURKB proteins are more-closer to each other with largest identity (54.3%) and similarity (63.4%) and, smallest gap (28.3%) compares to any other pair of HubGs (S2 File for MSA, phylogenetic tree, identity, similarity, score and gaps). The binding affinity scores of these two proteins were found significantly larger and almost same with respect to our suggested drug molecules (Fig 5A). On the other hand, we also observed that proteins OAS1 and FBXL3 are second more-closer to each other with larger identity (28.7%) and similarity (37.0%) and, smaller gap (52.7%) compares to any other pair of the rest HubGs. The binding affinity scores for these two proteins were also larger and almost similar against our suggested drug molecules. The MD simulation-based MM-PBSA analysis showed the average binding free energy for AURKA and AURKB are almost similar (58.5 kcal/mol & 53.92 kcal/mol) but far different from OAS1 (-6.834 kcal/mol). Thus, the molecular signatures and potential repurposable drug agents that we have identified in this study may serve as valuable resources for wet-lab validation and the development of an effective treatment plan against SARS-CoV-2 infections.

## Conclusions

This study suggested SARS-CoV-2 infection causing core genes (*REL*, *AURKA*, *AURKB*, *FBXL3*, *OAS1*, *STAT4*, *MMP2* and *IL6*) by highlighting their key transcriptional regulators (SRF, PBX1, MEIS1, ESR1 and MYC) and post-transcriptional regulators (hsa-miR-106b-5p, hsa-miR-20b-5p, hsa-miR-93-5p, hsa-miR-106a-5p and hsa-miR-20a-5p). To explore the effective drugs for SARS-CoV-2 infections by the molecular docking analysis, core gene mediated proteins and five TFs proteins were considered as the receptors. Based on our computational analysis, we nominated top-ranked 10 candidate drugs (Nilotinib, Tegobuvir, Digoxin, Proscillaridin, Olysio, Simeprevir, Hesperidin, Oleanolic Acid, Naltrindole, and Danoprevir) that showed the highest docking scores, indicating their favorable binding affinity with the receptors. Then we validated the suggested drug molecules against the state-of-the-art alternatives publicly available top-ranked 8 independent receptors (CASP3, CXCL8, ICAM1, IL6, NFKBIA, STAT1, TNF and IRF7) by molecular docking and found their significant binding affinities. Finally, we examined the stability of top-ranked three receptor-ligand complexes (AURKA vs. Nilotinib, AURKB vs. Tegobuvir, OAS1 vs. Proscillaridin) by computing the RMSD scores and binding free energies through the 100 ns MD-simulation based MM-PBSA approach, and observed their stable performance. In this regard, this study might open up a new gateway to explore more effective drug molecules computationally against SARS-CoV-2 infections. Thus, the outputs of this study might be useful inputs for wet-lab experiment to make a proper treatment plan against SARS-CoV-2 infections.

## Supporting information

S1 Table Collection of 177 meta drug agents by literature review. (DOCX)

S2 Table Targeted protein list from different published literature. (DOCX)

S1 File (I) Binding score of the interaction of targeted proteins with targeted drugs. (II) Binding score of the interaction of published proteins with proposed drugs and published drugs.
(XLSX)

S2 File (I) Multiple Sequence Alignment (MSA) Results for HubGs. (II) Identity, Similarity, Gap and Score matrices based on the alignment results of HubGs. (III) Phylogenetic tree of HubGs.
(XLSX)
