# Supplementary material for: Identification of host genomic biomarkers from multiple transcriptomics datasets for diagnosis and therapies of SARS-CoV-2 infections
Source: PLoS One. 2023 Mar 13;18(3):e0281981. doi: 10.1371/journal.pone.0281981 (PMC10010564; doi:10.1371/journal.pone.0281981)
Supplement: S1 Table — (DOCX) [file pone.0281981.s001.docx]

| **Table S1.** Collection of 177 meta drug agents against SARS-CoV-2 infection by the literature review**.** | |
| --- | --- |
| **FDA authorized anti-viral drugs** | |
| **Published Articles** | **Suggested Drug** |
| Beck et al. [1] | 5-nonyloxytryptamine, Abacavir sulfate, Abacavir, Acetylcholine Chloride, Acyclovir, Adefovir Dipivoxil, Amprenavir (agenerase), Apixaban, Asunaprevir (BMS-650032), Atazanavir sulfate (BMS-232632-05), Atazanavir, Atropine, avermectin, Batimastat, Boceprevir, bosutinib, Cidofovir, Cyclosporine, dacinostat, Daclatasvir (BMS-790052), danoprevir, Daptomycin, Darunavir, demecarium, Difloxacin HCl, dinoprostone, efavirenz, elvitegravir, Entecavir Hydrate, entecavir, eprosartan, Etomidate, everolimus, Famciclovir, foxy-5, Ganciclovir, indinavir, ivermectin, Leuprolide Acetate, lisuride, lopinavir, Methscopolamine, mupirocin, naltrindole, Nelfinavir Mesylate, nelfinavir, nevirapine, Octreotide acetate, oligomycin-a, Oseltamivir acid, Oseltamivir phosphate, Oseltamivir, Otilonium Bromide, Penciclovir, Peramivir Trihydrate, Peramivir, Pimecrolimus, prostaglandin, Radotinib(IY-5511), raltegravir, Rapamycin (Sirolimus), Remdesivir, ribavirin, Rifabutin, Rilpivirine, Ritonavir, Rupatadine Fumarate, Saquinavir mesylate, saquinavir, saracatinib, scopolamine, Sildenafil Citrate, sirolimus, somatostatin, Tacrolimus (FK506), Telaprevir (VX-950), temsirolimus, Tenofovir Disoproxil Fumarate, tenofovir, thiostrepton, Tigecycline, Tiotropium Bromide, torin-2, trichostatin-a, Valaciclovir HCl, valaciclovir, Valganciclovir HCl, Zanamivir, zolmitriptan, PHA-665752 |
| **Transcriptomic Drug** | |
| **Published Articles** | **Suggested Drug** |
| Ruan et al., 2021[2] | Nilotinib, Saquinavir, Tipranavir, Lonafarnib, Tegobuvir, Olysio, Filibuvir, and Cepharanthine |
| Gurudeeban Selvaraj et al. 2021[3] | Wortmannin |
| Tasnimul Alam Taz et al. 2020 [4] | MIGLITOL CTD 00002031, CHEMBL55802 CTD 00003118, Hesperidin CTD 00006087, Cytochalasin D CTD 00007076, Prolinedithiocarbamate CTD 00002658, Parthenolide CTD 00000087, FEXOFENADINE HYDROCHLORIDE CTD 00003191, Hydroxytyrosol CTD 00000267, Antimycin A CTD 00005427, Anacardic acid C15:3 CTD 00003117, |
| Mohammad Ali Moni et al. 2020 [5] | Cytochalasin D, 1′-acetoxychavicol acetate, Atorvastatin, Proline dithiocarbamate, Dicumarol, Oleanolic acid. |
| Islam et al. 2020 [6] | SYK-inhibitor, Radicicol, Dabrafenib, AT-7519, Dasatinib, Lovastatin, Thiostrepton, Linifanib, JNK–IN–5A, Withaferin-A. |
| Ge C et al 2020 [7] | Astragaloside IV |
| Aishwarya et al. 2020 [8] | F-1566-0341, Digoxin, Proscillaridin, Linifanib |
| Tao et. al., 2020 [9] | Quercetin, Kaempferol, Beta-sitosterol, Stigmasterol, Isorhamnetin, Baicalein, Naringenin, Formononetin |
| Han et. al, 2020 [10] | Quercetin, Luteolin |
| Li Zhonglin et al, 2020[11] | Podophyllotoxin, Amantadine, Thioperamide, Monensin, Vancomycin, Etiocholanolone, Acyclovir, Isoflupredone, Heptaminol,Chenodeoxycholic acid, Podophyllotoxin, Atractyloside, Adiphenine, Monensin, Lisuride |
| Fangzhou Liu_2021[12] | Matrine |
| Zulkar Nain et al. 2020 [13] | Antibiotic K-252A,Cabozantinib, Amuvatinib, Crizotinib, SGX-523, [888719-03-7](https://www.ncbi.nlm.nih.gov/pcsubstance/?term=%22888719-03-7%22%5bCompleteSynonym%5d%20AND%2021081761%5bStandardizedCID%5d), CHEMBL527066, [CHEMBL503090](https://www.ncbi.nlm.nih.gov/pcsubstance/?term=%22CHEMBL503090%22%5bCompleteSynonym%5d%20AND%2011560856%5bStandardizedCID%5d), SCHEMBL15322421, CHEMBL462712, [rac-crizotinib](https://www.ncbi.nlm.nih.gov/pcsubstance/?term=%22rac-crizotinib%22%5bCompleteSynonym%5d%20AND%2011597571%5bStandardizedCID%5d), [CHEMBL561660](https://www.ncbi.nlm.nih.gov/pcsubstance/?term=%22CHEMBL561660%22%5bCompleteSynonym%5d%20AND%2025229537%5bStandardizedCID%5d), Crizotinib, Cabozantinib. |
| Suresh Kumar _et al. 2020 [14] | Chloroquine, lenalidomide, Penicillin, Pentoxifylline, Thalidome, Sorafenib,  Paclitaxel, Rapamycin, Cortisol, Statins |
| Yi-Wei Zhu et al. 2020 [15] | Quercetin, Kaempferol, bsitosterol, Isorhamnetin, Naringenin, Luteolin, (þ)-catechin, Delphinidin, aloe-Emodin, Baicalein and Irisolidone |
| Zhen-Zhen [16] | berberine/NIT-X |

**References**

1. Beck BR, Shin B, Choi Y, Park S, Kang K. Predicting commercially available antiviral drugs that may act on the novel coronavirus (SARS-CoV-2) through a drug-target interaction deep learning model. Comput Struct Biotechnol J. 2020;18. doi:10.1016/j.csbj.2020.03.025

2. Ruan Z, Liu C, Guo Y, He Z, Huang X, Jia X, et al. SARS-CoV-2 and SARS-CoV: Virtual screening of potential inhibitors targeting RNA-dependent RNA polymerase activity (NSP12). J Med Virol. 2021;93. doi:10.1002/jmv.26222

3. Selvaraj G, Kaliamurthi S, Peslherbe GH, Wei D-Q. Identifying potential drug targets and candidate drugs for COVID-19: biological networks and structural modeling approaches. F1000Research. 2021;10: 127. doi:10.12688/f1000research.50850.1

4. Taz TA, Ahmed K, Paul BK, Kawsar M, Aktar N, Mahmud SMH, et al. Network-based identification genetic effect of SARS-CoV-2 infections to Idiopathic pulmonary fibrosis (IPF) patients. Brief Bioinform. 2020;00: 1–13. doi:10.1093/bib/bbaa235

5. Moni MA, Quinn JMW, Sinmaz N, Summers MA. Gene expression profiling of SARS-CoV-2 infections reveal distinct primary lung cell and systemic immune infection responses that identify pathways relevant in COVID-19 disease. Brief Bioinform. 2020;00: 1–14. doi:10.1093/bib/bbaa376

6. Islam T, Rahman MR, Aydin B, Beklen H, Arga KY, Shahjaman M. Integrative transcriptomics analysis of lung epithelial cells and identification of repurposable drug candidates for COVID-19. Eur J Pharmacol. 2020;887: 173594. doi:10.1016/j.ejphar.2020.173594

7. Ge C, He Y. In Silico Prediction of Molecular Targets of Astragaloside IV for Alleviation of COVID-19 Hyperinflammation by Systems Network Pharmacology and Bioinformatic Gene Expression Analysis. Front Pharmacol. 2020;11. doi:10.3389/fphar.2020.556984

8. Aishwarya S, Gunasekaran K, Margret AA. Computational gene expression profiling in the exploration of biomarkers, non-coding functional RNAs and drug perturbagens for COVID-19. J Biomol Struct Dyn. 2020;0: 1–16. doi:10.1080/07391102.2020.1850360

9. Tao Q, Du J, Li X, Zeng J, Tan B, Xu J, et al. Network pharmacology and molecular docking analysis on molecular targets and mechanisms of Huashi Baidu formula in the treatment of COVID-19. Drug Dev Ind Pharm. 2020;46: 1–9. doi:10.1080/03639045.2020.1788070

10. Han L, Wei XX, Zheng YJ, Zhang LL, Wang XM, Yang HY, et al. Potential mechanism prediction of Cold-Damp Plague Formula against COVID-19 via network pharmacology analysis and molecular docking. Chinese Med (United Kingdom). 2020;15. doi:10.1186/s13020-020-00360-8

11. Li Z, Yang L. Underlying Mechanisms and Candidate Drugs for COVID-19 Based on the Connectivity Map Database. Front Genet. 2020;11. doi:10.3389/fgene.2020.558557

12. Liu F, Li Y, Yang Y, Li M, Du Y, Zhang Y, et al. Study on mechanism of matrine in treatment of COVID-19 combined with liver injury by network pharmacology and molecular docking technology. Drug Deliv. 2021;28. doi:10.1080/10717544.2021.1879313

13. Nain Z, Rana HK, Liò P, Islam SMS, Summers MA, Moni MA. Pathogenetic profiling of COVID-19 and SARS-like viruses. Brief Bioinform. 2021;22. doi:10.1093/bib/bbaa173

14. Kumar S. COVID-19: A drug repurposing and biomarker identification by using comprehensive gene-disease associations through protein-protein interaction network analysis. Preprints. 2020. doi:10.20944/preprints202003.0440.v1

15. Zhu YW, Yan XF, Ye TJ, Hu J, Wang XL, Qiu FJ, et al. Analyzing the potential therapeutic mechanism of Huashi Baidu Decoction on severe COVID-19 through integrating network pharmacological methods. J Tradit Complement Med. 2021;11. doi:10.1016/j.jtcme.2021.01.004

16. Wang ZZ, Li K, Maskey AR, Huang W, Toutov AA, Yang N, et al. A small molecule compound berberine as an orally active therapeutic candidate against COVID-19 and SARS: A computational and mechanistic study. FASEB J. 2021;35. doi:10.1096/fj.202001792R
