# Supplementary material for: Identification of host genomic biomarkers from multiple transcriptomics datasets for diagnosis and therapies of SARS-CoV-2 infections
Source: PLoS One. 2023 Mar 13;18(3):e0281981. doi: 10.1371/journal.pone.0281981 (PMC10010564; doi:10.1371/journal.pone.0281981)
Supplement: S2 Table — (DOCX) [file pone.0281981.s002.docx]

| **Table S2.** Targeted protein list from different published literature. | | | |
| --- | --- | --- | --- |
| **Different key protein lists for SARS-CoV-2 infection published by different research articles in different international reputed journals** | | | |
| **Articles** | **Hub-proteins** | **Common hub genes with at least 3 articles** | **Common hub genes with at least 4 articles** |
| **Xie et al.** [1] | CXCL1, CXCL2, TNF, NFKBIA, CSF2, TNFAIP3, IL6, CXCL3, CCL20, ICAM1 | IL6, TNF, CXCL8, ICAM1, IRF7, NFKBIA, STAT1, CASP3 | IL6, TNF, VEGFA |
| **Jung et al.** [2] | GATA4, ID2, MAFA, NOX4, PTBP1, SMAD3, TUBB1, WWOX |  |  |
| **Vastrad et al.** [3] | TP53, HRAS, CTNNB1, FYN, ABL1, STAT3, STAT1, JAK2, C1QBP, XBP1, BST2, CD99, IFI35, MAPK11, RELA, LCK, KIT, EGR1, IL20, ILF3, CASP3, IL19, ATG7, GPI, S1PR1 |  |  |
| **Prasad et al.** [4] | STAT1, IRF7, IFIH1, MX1, ISG15, IFIT3, OAS2, DDX58, IRF9, IFIT1,  OAS1, OAS3, DDX60, OASL, IFIT2 |  |  |
| **Selvaraj et al.**[5] | MYC, HDAC9, NCOA3, CEBPB, VEGFA, BCL3, SMAD3, SMURF1, KLHL12, CBL, ERBB4, CRKL |  |  |
| **Satu et al.** [6] | MARCO, VCAN, ACTB, LGALS1, HMOX1, TIMP1, OAS2, GAPDH, MSH3, FN1, NPC2, JUND, GPNMB, SYTL2, CASP1, S100A8, MYO10, IGFBP3, APCDD1, COL6A3, FABP5, PRDX3, CLEC1B, DDIT4, CXCL10, CXCL8 |  |  |
| **Taz et al.** [7] | VEGFA, AKT1, MMP9, ICAM1, CD44 |  |  |
| **Moni et al.** [8] | MX1, IRF7, BST2 |  |  |
| **Islam et al.** [9] | BIRC3, ICAM1, IRAK2, MAP3K8, S100A8, SOCS3, STAT5A, TNF, TNFAIP3, TNIP1 |  |  |
| **Zhou et al.** [10] | JUN, XPO1, NPM1, HNRNPA1 |  |  |
| **Ge et al.** [11] | MMP13, NLRP3, GBP1, ADORA2A, PTAFR, TNF, MLNR, IL1B, NFKBIA, ADRB2, IL6 |  |  |
| **Aishwarya et al.**[12] | IGF2, HINT1, MAPK10, SGCE, HDAC5, SGCA, SGCB, CFD, ITSN1, EHMT2, CLU, ISLR, PGM5, ANK2, HDAC9, SYT11, MDH1, SCCPDH, SIRT6, DTNA, FN1, ARRB1, MAGED2, TEX264, VEGFC, HK2, TXNL4A, SLC16A3, NUDT21, TRA2B, HNRNPA1, CDC40, THOC1, PFKFB3 |  |  |
| **Saxena, *et al*** [13] | STAP1, CASP5, FDCSP, CARD17, ST20, AKR1B10, CLC, KCNJ2-AS1, RNASE2 , FLG |  |  |
| **Tao et al.** [14] | MAPK3, MAPK8, TP53, CASP3, IL6, TNF, MAPK1, CCL2, PTGS2 |  |  |
| **Zhang et. al.** [15] | CXCL10, ISG15, DDX58, MX2, OASL, STAT1, RSAD2, MX1, IRF7, OAS1 |  |  |
| **Han L et. al,** [16] | IL6 , TNF ,IL10, MAPK8,MAPK3,CXCL8,CASP3,PTGS2, TP53, MAPK1 |  |  |
| **Wang et al.** [17] | CXCL8, CXCL1, CXCL2, CCL20, CSF2 |  |  |
| **Gu et al.** [18] | NFKBIA, C3, CCL20 |  |  |
| **Nan et al.** [19] | ALB, CXCL8, FGF2, IL6, INS, MMP2, MMP9, PTGS2, STAT3, VEGFA |  |  |
| **Gu et al.** [20] | CDC20, NCBP1, POLR2D, DYNLL1, FBXW5, LRRC41, FBXO21, FBXW9, FBXO44, FBXO6 |  |  |
| **Sardar et al.** [21] | HMOX1, DNMT1, PLAT, GDF1, ITGB1 |  |  |
| **Gu et al.** [22] | FLOC, DYNLL1, FBXL3 and FBXW11 |  |  |
| **Vastrad et al.** [23] | VCAM1, IKBKE, STAT1, IL7R, ISG15, E2F1, ZBTB16, TFAP4, ATP6V1B1 and APBB1 |  |  |
| **B. Vastrad et al** [24] | CBL, ISG15, NEDD4, PML, REL, CTNNB1, ERBB2, JUN, RPS8 and STUB1 |  |  |

**References:**

1. Xie T-A, Han M-Y, Su X-R, Li H-H, Chen J-C, Guo X-G. Identification of Hub genes associated with infection of three lung cell lines by SARS-CoV-2 with integrated bioinformatics analysis. Journal of cellular and molecular medicine. 2020;24: 12225.

2. Oh JH, Tannenbaum A, Deasy JO. Identification of biological correlates associated with respiratory failure in COVID-19. BMC Medical Genomics. 2020;13: 1–6.

3. Vastrad B, Vastrad C, Tengli A. Identification of potential mRNA panels for severe acute respiratory syndrome coronavirus 2 (COVID-19) diagnosis and treatment using microarray dataset and bioinformatics methods. 3 Biotech. 2020;10: 1–65.

4. Prasad K, Khatoon F, Rashid S, Ali N, AlAsmari AF, Ahmed MZ, et al. Targeting hub genes and pathways of innate immune response in COVID-19: a network biology perspective. International journal of biological macromolecules. 2020;163: 1–8.

5. Selvaraj G, Kaliamurthi S, Peslherbe GH, Wei D-Q. Identifying potential drug targets and candidate drugs for COVID-19: biological networks and structural modeling approaches. F1000Research. 2021;10.

6. Satu MS, Khan MI, Rahman MR, Howlader KC, Roy S, Roy SS, et al. Diseasome and comorbidities complexities of SARS-CoV-2 infection with common malignant diseases. Briefings in Bioinformatics. 2021;22: 1415–1429.

7. Taz TA, Ahmed K, Paul BK, Kawsar M, Aktar N, Mahmud SH, et al. Network-based identification genetic effect of SARS-CoV-2 infections to Idiopathic pulmonary fibrosis (IPF) patients. Briefings in Bioinformatics. 2021;22: 1254–1266.

8. Moni MA, Quinn JM, Sinmaz N, Summers MA. Gene expression profiling of SARS-CoV-2 infections reveal distinct primary lung cell and systemic immune infection responses that identify pathways relevant in COVID-19 disease. Briefings in bioinformatics. 2021;22: 1324–1337.

9. Islam T, Rahman MR, Aydin B, Beklen H, Arga KY, Shahjaman M. Integrative transcriptomics analysis of lung epithelial cells and identification of repurposable drug candidates for COVID-19. European Journal of Pharmacology. 2020;887: 173594.

10. Zhou Y, Hou Y, Shen J, Huang Y, Martin W, Cheng F. Network-based drug repurposing for novel coronavirus 2019-nCoV/SARS-CoV-2. Cell discovery. 2020;6: 1–18.

11. Ge C, He Y. In Silico prediction of molecular targets of Astragaloside IV for alleviation of COVID-19 Hyperinflammation by systems network pharmacology and Bioinformatic gene expression analysis. Frontiers in pharmacology. 2020;11: 1494.

12. Aishwarya S, Gunasekaran K, Margret AA. Computational gene expression profiling in the exploration of biomarkers, non-coding functional RNAs and drug perturbagens for COVID-19. Journal of Biomolecular Structure and Dynamics. 2020; 1–16.

13. Saxena A, Chaudhary U, Bharadwaj A, Wahi N, Kalli JR, Gupta S, et al. A lung transcriptomic analysis for exploring host response in COVID-19. J Pure Appl Microbiol. 2020;14: 1077–1081.

14. Tao Q, Du J, Li X, Zeng J, Tan B, Xu J, et al. Network pharmacology and molecular docking analysis on molecular targets and mechanisms of Huashi Baidu formula in the treatment of COVID-19. Drug development and industrial pharmacy. 2020;46: 1345–1353.

15. Zhang N, Zhao YD, Wang XM. CXCL10 an important chemokine associated with cytokine storm in COVID-19 infected patients. Eur Rev Med Pharmacol Sci. 2020;24: 7497–7505.

16. Han L, Wei X-X, Zheng Y-J, Zhang L-L, Wang X-M, Yang H-Y, et al. Potential mechanism prediction of Cold-Damp Plague Formula against COVID-19 via network pharmacology analysis and molecular docking. Chinese Medicine. 2020;15: 1–16.

17. Wang Z, Jiang C, Zhang X, Zhang Y, Ren Y, Gao X. Identification of Key Genes and Pathways in SARS-CoV-2 Infection using Bioinformatics Analysis. 2020.

18. Gu H, Yuan G. Identification of potential key genes for SARS-CoV-2 infected human bronchial organoids based on bioinformatics analysis. bioRxiv. 2020.

19. Nana KS, Karuppanan K, Kumar S. Identification of common key genes and pathways between Covid-19 and lung cancer by using protein-protein interaction network analysis. bioRxiv. 2021.

20. Gu H, Yuan G. Identification of key genes and pathways in the hPSC-derived lungs infected by the SARS-CoV-2. 2020.

21. Sardar R, Satish D, Gupta D. Identification of novel SARS-CoV-2 drug targets by host microRNAs and transcription factors co-regulatory interaction network analysis. Frontiers in Genetics. 2020;11: 1105.

22. Gu H, Yuan G. Identification of key genes in SARS-CoV-2 patients on bioinformatics analysis. bioRxiv. 2020.

23. Vastrad B, Vastrad C, Kotturshetti I. Integrated bioinformatics analysis for the screening of hub genes and therapeutic drugs in severe acute respiratory syndrome corona virus 2 infection/COVID 19. 2020.

24. Vastrad B, Vastrad C, Tengli A. Bioinformatics analyses of significant genes, related pathways, and candidate diagnostic biomarkers and molecular targets in SARS-CoV-2/COVID-19. Gene Reports. 2020;21: 100956.
